# *Shigella* IpaH Family Effectors as a Versatile Model for Studying Pathogenic Bacteria

**DOI:** 10.3389/fcimb.2015.00100

**Published:** 2016-01-06

**Authors:** Hiroshi Ashida, Chihiro Sasakawa

**Affiliations:** ^1^Division of Bacterial Infection Biology, Institute of Medical Science, University of TokyoTokyo, Japan; ^2^Nippon Institute for Biological ScienceTokyo, Japan; ^3^Medical Mycology Research Center, Chiba UniversityChiba, Japan

**Keywords:** NF-kB, *Shigella*, effector, ubiquitin, E3 ligase

## Abstract

*Shigella* spp. are highly adapted human pathogens that cause bacillary dysentery (shigellosis). Via the type III secretion system (T3SS), *Shigella* deliver a subset of virulence proteins (effectors) that are responsible for pathogenesis, with functions including pyroptosis, invasion of the epithelial cells, intracellular survival, and evasion of host immune responses. Intriguingly, T3SS effector activity and strategies are not unique to *Shigella*, but are shared by many other bacterial pathogens, including *Salmonella, Yersinia*, and enteropathogenic *Escherichia coli* (EPEC). Therefore, studying *Shigella* T3SS effectors will not only improve our understanding of bacterial infection systems, but also provide a molecular basis for developing live bacterial vaccines and antibacterial drugs. One of *Shigella* T3SS effectors, IpaH family proteins, which have E3 ubiquitin ligase activity and are widely conserved among other bacterial pathogens, are very relevant because they promote bacterial survival by triggering cell death and modulating the host immune responses. Here, we describe selected examples of *Shigella* pathogenesis, with particular emphasis on the roles of IpaH family effectors, which shed new light on bacterial survival strategies and provide clues about how to overcome bacterial infections.

## Introduction

The interplay between enteric pathogens and the gastrointestinal (GI) tract is a critical determinant of the fate of bacterial infection and disease progression. *Shigella* spp. are highly adapted human pathogens that cause shigellosis, a disease that provokes severe bloody and mucous diarrhea, which still remains a major public health concern in developing countries. No safe and effective *Shigella* vaccine currently exists, and antibiotic-resistant bacteria have recently been observed, increasing the threat of outbreaks due to lack of effective treatments (Barry et al., 2013). Therefore, there is an urgent need to understand the interplay between bacterial infection and host cellular and immune responses in order to develop safe and effective *Shigella* vaccines or novel treatment.

After ingestion via the fecal–oral route, *Shigella* preferentially invade the intestinal epithelium via M cells overlying the follicle-associated epithelium. Once *Shigella* are endocytosed by M cells, they enter resident macrophages and induce inflammatory cell death (Wassef et al., 1989; Perdomo et al., 1994). After being released from dying macrophages, the bacteria invade surrounding epithelial cells, escape from the vacuole, and multiply within the cytoplasm (Zychlinsky et al., 1992). Subsequently, *Shigella* move by inducing actin polymerization at one pole of the bacterium, thereby spreading to neighboring cells (Suzuki et al., 1998, 2000; Egile et al., 1999). Thus, shigellosis is the consequence of multiple pathogenic events, including macrophage cell death, invasion of and multiplication within epithelial cells, cell-cell spread, and severe intestinal inflammation.

The infection strategies of *Shigella* depend on the delivery of bacterial virulence proteins, called “effectors,” into host cells via the type III secretion system (T3SS) (Carayol and Tran Van Nhieu, 2013; Ashida et al., 2015). The effectors, which are delivered into both myeloid cells (macrophages, dendritic cells, T, and B cells) and nonmyeloid cells (epithelial cells), mimic or subvert host cellular functions, allowing *Shigella* to colonize the intestinal epithelium. The diverse roles of effectors include bacterial invasion into cells, intracellular survival and multiplication, maintenance of an infectious foothold, and modulation of the host immune response. Recent studies showed that many T3SS effectors have novel and specific enzymatic activities distinct from those of mammalian enzymes, e.g., phosphothreonine lyase, deamidase, and E3 ubiquitin ligase (Li et al., 2007; Rohde et al., 2007; Sanada et al., 2012). Moreover, T3SS effector activity and function is not specific to *Shigella* but is shared by other enteric pathogens, including *Salmonella, Yersinia*, and EPEC. Therefore, the study of effector functions will improve our understanding of bacterial infection mechanisms and facilitate development of novel vaccines or drugs to overcome bacterial infection. *Shigella* is a good model for such studies because it has many effectors, some of which are common to other pathogens, and many of its strategies for infection are widely conserved among other enteric bacterial pathogens. In this review, we will highlight the role of one class of *Shigella* T3SS effector, the IpaH family.

## IpaH family effectors

*Shigella* possesses 12 *ipaH* genes, which reside on both the large plasmid and the chromosome. The encoded IpaH proteins are injected into host cells via the T3SS (Ashida et al., 2007). IpaH family proteins contain N-terminal leucine-rich repeats (LRRs) and have E3 ubiquitin ligase activity in their conserved C-terminal regions (Rohde et al., 2007; Ashida et al., 2014a). Ubiquitination is an important post-translational modification that regulates several cellular functions, including cell signaling, protein degradation, transcription, and endocytosis (Pickart, 2001). Ubiquitination is accomplished via a series of reactions catalyzed by a multienzymatic cascade: E1 (ubiquitin-activating enzyme), E2 (ubiquitin-conjugating enzyme), and E3 (ubiquitin ligase). The ubiquitination cascade starts with ATP-dependent activation of ubiquitin via formation of a thioester linkage between the carboxyl-terminal Gly of ubiquitin and a Cys of E1. Activated ubiquitin is transferred to the active-site Cys of E2, and finally E3 ligase mediates the transfer of ubiquitin from the E2 to specific substrate proteins (mainly via substrate Lys residues). E3 ligases can be categorized into two groups based on their structures and functions: HECT (Homologous to the E6-AP Carboxyl Terminus)-type and RING (Really Interesting New Gene)/U-box-type. HECT-type E3 ligases catalyze ubiquitin transfer by accepting ubiquitin from E2 via formation of a thioester bond with their catalytic cysteine residue, and then transfer ubiquitin to their target substrates. On the other hand, RING/U-box-type E3 ligases catalyze direct ubiquitin transfer by acting as scaffold molecules to bind and recruit the E2-ubiquitin complex, and then directly transfer ubiquitin from E2 to E3-bound substrates. Recent reports showed that some bacterial pathogens deliver several types of T3SS effectors with E3 ligase activity (Ashida et al., 2014a). Because the ubiquitination pathway is absent in bacteria, bacteria might deliver E3 ligase effectors and hijack the mammalian ubiquitination pathway in order to counteract host responses. IpaH family proteins are widely conserved among animal and plant pathogens, including *Shigella* (IpaH), *Salmonella* (SspH1, SspH2, and SlrP), *Edwardsiella, Bradyrhizobium, Rhizobium*, and some *Pseudomonas* species, illustrating the importance of these effectors in bacterial infection. Although IpaH family proteins have E3 ubiquitin ligase activity and their C-terminal domains contain a single conserved Cys that form a Cys-ubiquitin intermediate similar to that of HECT-type ligases, the catalytic domains of IpaH family members differ at the sequence and structural levels from eukaryotic E3 ubiquitin ligases. Consequently, IpaH family proteins are now considered to constitute a new class of E3 ubiquitin ligases, NEL (Novel E3 ligase), distinct from typical RING-, and HECT-types of E3 ubiquitin ligases (Singer et al., 2008; Zhu et al., 2008; Quezada et al., 2009). Intriguingly, IpaH family proteins exhibit auto-inhibition to prevent ubiquitination of unintended proteins. Specifically, the LRR domain sequesters and masks the catalytic Cys residue in the C-terminal region prior to substrate binding. Once the LRR binds to substrate proteins, a conformational change occurs, releasing the auto-inhibition of IpaH E3 ligase activity (Quezada et al., 2009; Chou et al., 2012; Keszei et al., 2014).

Although IpaH family proteins are highly similar to one another, the sequences of their LRR regions, regarded as substrate recognition sites, and subcellular localizations (e.g., nucleus, cytoplasm, or plasma membrane) are different. These observations imply that each IpaH family protein has a specific host target protein due to its unique subcellular localization and substrate recognition domain, and thus makes a distinct contribution to promotion of bacterial pathogenesis. Consistent with this, recent studies have revealed the role of IpaH family members in disabling distinct target proteins in host cells.

## *Shigella* IpaH effectors target NF-κB signaling

**Figure 1:**
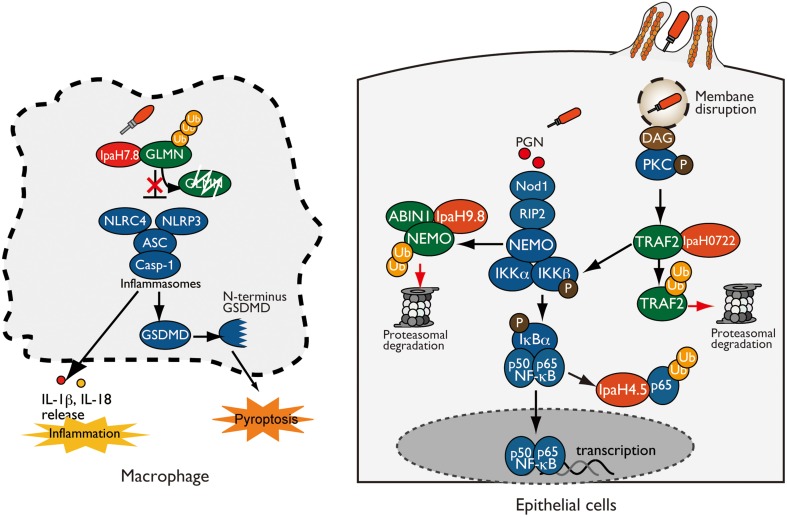
***Shigella* IpaH effectors target host substrates for ubiquitination**. (**left:** Macrophage) IpaH7.8 interacts with and ubiquitinates GLMN, leading to its proteasome-dependent degradation. Degradation of GLMN abolishes its ability to inhibit inflammasomes, resulting in NLRP3/NLRC4 inflammasome activation and pyroptosis. (**right:** Epithelial cell) IpaH0722, IpaH9.8, and IpaH4.5 target and ubiquitinate TRAF2, NEMO, and p65, respectively, which undergo proteasome degradation, resulting in inhibition of NF-κB.

The host innate immune system can detect markers of *Shigella* infection as pathogen-associated molecular patterns (PAMPs) and danger-associated molecular patterns (DAMPs), leading to severe inflammatory colitis aimed at preventing and clearing bacterial infection. However, many enteric bacterial pathogens, including *Shigella*, are able to optimize the host inflammatory responses to allow pathogen survival in such inflamed environments by delivering a subset of T3SS effector proteins (Rahman and McFadden, 2011; Ashida et al., 2015). Because the nuclear factor κB (NF-κB) plays a pivotal role in the bacteria-induced inflammatory response, *Shigella* deliver effectors that target and inhibit NF-κB signaling to modulate the host inflammatory response (Ashida et al., 2015; Rahman and McFadden, 2011). Recognition of PAMPs or DAMPs by various sensors, including Toll-like receptors (TLRs) and Nod-like receptors (NLRs), and subsequent signal transduction through Myd88, TRAF, RIP, TAK1, and TAB2/3 trigger the activation of IKK kinase complex. Once the IKK complex is activated, it phosphorylates IκB (inhibitor of NF-κB), leading to its ubiquitination and proteasome degradation. NF-κB then translocates to the nucleus and activates the transcription of proinflammatory cytokines (Hayden and Ghosh, 2012). Because ubiquitination is an essential aspect of this regulatory mechanism, *Shigella* deliver three IpaH effectors (IpaH9.8, IpaH0722, and IpaH4.5) that subvert NF-κB activation by manipulating the host ubiquitin system (Figure 1). After *Shigella* invasion of epithelial cells, the host sensor Nod1 recognizes peptidoglycans released from *Shigella* as PAMPs, triggering NF-κB activation. IpaH9.8 preferentially prevents Nod1-dependent NF-κB activation. Specifically, IpaH9.8 interacts with NEMO/IKKγ, which is critical for downstream activation of NF-κB, and targets NEMO (lysines 309 and 321 residues) for ubiquitination. IpaH9.8 also interacts with ABIN-1, a ubiquitin-binding adaptor protein and bridge IpaH9.8-NEMO, to further promote polyubiquitination of NEMO. Ubiquitination of NEMO by IpaH9.8 leads to proteasomal degradation, thereby reducing NF-κB activation (Ashida et al., 2010). Consistent with this model, the level of NEMO protein and *Shigella*-induced NF-κB activation were decreased by IpaH9.8 E3 ligase activity in NEMO-WT cells, but not in cells stably expressing NEMO-K309R/K321R, a mutant lacking the IpaH9.8-mediated ubiquitination site. The reduction in NF-κB activation due to the activity of this E3 ligase effector during *Shigella* infection results in downregulation of host inflammatory responses, such as cytokine production and neutrophil recruitment, and contributes to bacterial colonization in mouse models of lung infection (Ashida et al., 2010). In addition to NEMO, IpaH9.8 also binds to the host splicing factor U2AF35 and inhibits the U2AF35-dependent mRNA splicing reaction (Okuda et al., 2005). Because IpaH9.8 ubiquitinates U2AF35 *in vitro*, it has been proposed that IpaH9.8 diminishes the amount of U2AF35 and downregulates U2AF35-dependent pro-inflammatory gene expression (Seyedarabi et al., 2010).

Another IpaH family protein, IpaH0722 localizes to host cell plasma membrane via the modulation of *S*-palmitoylation at N-terminal Cys14 and Cys18 residues. IpaH0722 E3 ligase preferentially inhibits phorbol myristate acetate (PMA)-mediated NF-κB activation in NF-κB-luciferase reporter assays. Since PMA mimics the role of diacylglycerol (DAG) in the activation of the protein kinase C (PKC)-NF-κB pathway, IpaH0722 also inhibits PKC-mediated NF-κB activation (Ashida et al., 2013). Intriguingly, DAG is recognized as the danger signals that are generated by vacuolar membrane ruptures in *Shigella* infection. When *Shigella* invades epithelial cells, the bacteria are surrounded by a vacuolar membrane, but they rapidly disrupt this membrane and disseminate into the cytoplasm. At this step, *Shigella*-mediated vacuolar membrane ruptures are recognized as DAMPs, triggering NF-κB activation through the recruitment and activation of PKC. To counteract PKC–NF-κB activation, *Shigella* delivers IpaH0722. IpaH0722 interacts with TRAF2, a molecule downstream of PKC, and targets it for ubiquitination. TRAF2 ubiquitinated by IpaH0722 is degraded by the proteasome, thereby inhibiting PKC-mediated NF-κB activation (Ashida et al., 2013). In addition to IpaH9.8 and IpaH0722, *Shigella* also prevent NF-κB activation by delivering IpaH4.5, which targets the p65 subunit of the NF-κB complex targets it for ubiquitination *in vitro* (Wang et al., 2013).

In addition to the IpaH family, other *Shigella* effectors, such as OspG, OspI, and OspZ, also manipulate the ubiquitination system to inhibit NF-κB activation. OspG, which is homologous to EPEC NleH, is a serine/threonine kinase that binds to ubiquitin and ubiquitinated E2s to prevent phospho-IκBα ubiquitination, which is required for NF-κB activation (Kim et al., 2005; Zhou et al., 2013; Grishin et al., 2014; Pruneda et al., 2014). Another *Shigella* effector, OspI, has deamidase activity, which is shared by other effectors such as Cif from EPEC and CHBP from *Burkholderia pseudomallei*. OspI targets Ubc13, an E2 protein required for TRAF6 E3 ligase, and converts Gln-100 of Ubc13 to Glu-100, thereby inactivating its E2 activity. Inactivation of Ubc13 by OspI blocks TRAF6-dependent NF-κB signaling, thereby downregulating host inflammation (Sanada et al., 2012). OspZ from *S. flexneri* 6 and *S. boydii* blocks the nuclear translocation of NF-κB subunit p65, thereby inhibiting NF-κB activation (Newton et al., 2010). Although it remains unknown whether OspZ shares the same enzymatic activity, EPEC T3SS effector NleE has methyltransferase activity that specifically modifies zinc-finger cysteines of TAB2 and TAB3, ubiquitin-chain binding proteins involved in the NF-κB activation. Following cysteine methylation by NleE, TAB2, and TAB3 lose their zinc ions and ubiquitin-chain binding activities, making them unable to activate NF-κB (Zhang et al., 2011).

Furthermore, *Shigella* hijack epigenetic modification to modulate host innate immune responses. *Shigella* T3SS effector OspF, which is homologous to *Salmonella* SpvC and *Pseudomonas syringae* HopAl1, has a unique phosphothreonine lyase activity (Arbibe et al., 2007; Kramer et al., 2007; Li et al., 2007). OspF irreversibly dephosphorylates and inactivates MAPKs. This inactivation of MAPK further inhibits downstream phosphorylation of histone H3 at Ser10 at the promoters of a subset of innate immune genes, and promotes chromatin condensation, resulting in inhibition of transcriptional activation by masking NF-κB binding sites (Arbibe et al., 2007). Recent study further showed a novel function of OspF, which alters the activity of the chromatin reader Heterochromatin Protein 1γ (HP1γ) and reprograms host gene expression during *Shigella* infection via its phosphothreonine lyase activity (Harouz et al., 2014).

## *Shigella* IpaH effector targets inflammasomes

In addition to NF-κB signaling, *Shigella* use IpaH proteins to target inflammasome activation. As mentioned above, *Shigella* invade resident macrophages, disrupt the phagosome vacuole, disseminate into and multiply within the cytosol, and induce a form of inflammatory cell death called pyroptosis, which is accompanied by NLRP3 or NLRC4 inflammasome activation and leads to IL-1β and IL-18 secretion (Suzuki et al., 2007; Willingham et al., 2007; Ashida et al., 2011, 2014b; Davis et al., 2011; Suzuki et al., 2014a). Pyroptosis is a specialized form of cell death that is regulated by activated inflammatory caspases (caspase-1 or caspase-11). Caspase-1 is activated by canonical inflammasome (multiprotein complexes, such as NLRP3 or NLRC4 and ASC) via recognition of T3SS components, whereas caspase-11 directly binds to cytosolic LPS and forms non-canonical inflammasomes; both complexes trigger the release of proinflammatory cytokines (IL-1β and IL-18) and pyroptosis (Kayagaki et al., 2011, 2013; Shi et al., 2014). Recent studies show that activated caspase-1 and caspase-11 cleave GSDMD, and the cleaved N-terminal domain of GSDMD induces pyroptosis accompanied by cell lysis and release of cell contents (Kayagaki et al., 2015; Shi et al., 2015).

Although pyroptosis seems like a host defense system aimed at clearing bacterial infection, *Shigella* deliver IpaH7.8 to induce inflammasome activation and pyroptosis via its E3 ligase activity in order to escape from macrophages; otherwise, the bacteria will be killed (Figure 1) (Suzuki et al., 2014b). In a mouse model of lung infection, mice infected with the *Shigella* WT or Δ*ipaH7.8/WT* (IpaH7.8 WT complemented) strain exhibited severe macrophage cell death and IL-1β secretion, whereas those infected with the Δ*ipaH7.8* or Δ*ipaH7.8/CA* (E3 ligase-deficient mutant) strain did not. Notably, colonizing bacterial number was significantly higher in mice infected with the *Shigella* WT or Δ*ipaH7.8/WT* strain than in mice infected with Δ*ipaH7.8* or Δ*ipaH7.8/CA* mutants, illustrating the importance of IpaH7.8-mediated cell death in *Shigella* infection. IpaH7.8 interacts with GLMN, which acts either directly or indirectly as a negative regulator of the NLRP3/NLRC4 inflammasome, and targets it for ubiquitination. GLMN ubiquitinated by IpaH7.8 is subsequently degraded by the proteasome. GLMN degradation abolishes its inhibitory activity and thus triggers NLRP3/NLRC4 inflammasome activation and pyroptosis. To further support this idea, *Shigella*-induced cytotoxicity and IL-1β secretion levels were remarkably higher in macrophages derived from GLMN+∕− mice than in those from GLMN+∕+ mice. Furthermore, the number of internalized bacterial number was also higher in the lungs of GLMN+∕− mice (Suzuki et al., 2014b). Therefore, induction of pyroptosis by IpaH7.8 is more beneficial to *Shigella* than the host: specifically, it results in further invasion of surrounding epithelial cells and spread to neighboring cells, thereby promoting bacterial survival.

## Conclusion

The lack of a licensed vaccine and the emergence of antibiotic-resistant *Shigella* highlight the difficulty of controlling this pathogen and the need for new antimicrobial strategies. Studying the course of *Shigella* infection, especially the interaction between bacterial effectors and host cells, might provide a means to resolve this problem. In this report, we highlighted *Shigella* infection strategies mediated by IpaH family effectors, including NF-κB inhibition and macrophage killing. As mentioned above, IpaH family effectors are novel E3 ubiquitin ligases that are widely conserved among Gram-negative bacterial pathogens. The conservation of IpaH effectors among *Shigella* and bacterial pathogens, and their critical roles in pathogenesis, make them good targets for antibacterial drug or vaccine candidates. Recent studies show that each IpaH protein interacts with a specific host target protein via its LRR region, and then modulates the ubiquitination of that target. In addition to the *Shigella* IpaH family effectors, SspH1, SspH2, and SlrP from *Salmonella* target and ubiquitinate PKN1, SGT1, and Nod1, and thioredoxin, respectively (Haraga and Miller, 2006; Bernal-Bayard and Ramos-Morales, 2009; Bhavsar et al., 2013; Keszei et al., 2014). Although, the LRR regions of IpaH family effectors are slightly different, each protein contains a catalytic Cys residue that is required for transferring ubiquitin to substrate proteins. Therefore, an inhibitor that could block or mask the catalytic Cys residue of IpaH, or a conserved IpaH C-terminal-based vaccine, would provide broad coverage across against many bacterial pathogens. Therefore, the study of IpaH family effectors may lead to the development of a single drug that is effective against many infectious bacterial diseases.

### Conflict of interest statement

The authors declare that the research was conducted in the absence of any commercial or financial relationships that could be construed as a potential conflict of interest.
